# Nothing about us without us: harnessing local voices in shaping community-based adaptation in the Pacific

**DOI:** 10.1007/s11625-025-01638-2

**Published:** 2025-03-06

**Authors:** Hannah Turner, Briony Rogers, Sarah Kneebone, Diego Ramirez, Matthew French, Mere Jane Sawailau, Filise Volavola, Sholyn Baran, Kelera Matavesi, Orlando Newton, Maraia Batiota Luveniyali, Autiko Tela, Isoa Vakarewa

**Affiliations:** 1 Monash Sustainable Development Institute, Monash University, Melbourne, Australia; 2 Faculty of Art, Design and Architecture, Monash University, Melbourne, Australia; 3 Revitalising Informal Settlements and Their Environments (RISE)-Suva, Suva, Fiji; 4 Live and Learn, Suva, Fiji; 5 University of the South Pacific, Suva, Fiji; 6 Fiji National University, Suva, Fiji

**Keywords:** Climate adaptation, Flood protection, Community-based adaptation, Informal urban settlements, Pacific Island countries

## Abstract

As global temperatures rise, so does the frequency and intensity of severe weather events and their risks to people and assets. These risks are especially acute for Pacific Islanders in urban informal settlements, given their socio-economic vulnerabilities and limited political influence. There is growing awareness that national adaptation strategies may not fully meet the needs of these vulnerable communities, leading to a focus on community-led adaptation. However, these approaches are in their infancy and have been criticised for fostering paternalistic tendencies, prompting calls for external institutions to facilitate rather than direct community initiatives. This research utilises Photovoice, a method recognised for its cultural relevance and ability to amplify Indigenous and marginalised voices. It involves 42 households in Fiji's Greater Suva Urban Area, using resident-led photography and interviews to explore community-based flood adaptation. Through ethnographic content analysis and inductive coding, the study captures residents’ experiences and strategies, identifying over 31 unique adaptation measures and underscoring the importance of resources, social networks, traditional knowledge, beliefs, and leadership in enhancing adaptive capacity. The findings demonstrate the complexity of factors influencing adaptation, with resource availability and social capital being crucial. The study advocates for adaptive processes that are community driven, calling for a shift in research and funding to support these programmes in a flexible, responsive, and inclusive manner. It also highlights the need to understand community dynamics to prevent paternalistic practices and integrate local insights effectively, ensuring community self-determination in adaptation efforts.

## Introduction

Human-induced climate change has led to rising global temperatures, widespread changes in weather patterns, and increased risk of climate hazards (Government of Fiji 2017; Intergovernmental Panel on Climate Change [IPCC], 2022). Pacific Island countries (PICs) in particular are experiencing the devastating effects of sea-level rise, increasing temperatures, changes in precipitation patterns and increased frequency of climate hazards such as cyclones and tsunamis, exacerbating the threat of king tides, flooding, and coastal erosion, and posing a significant risk to urban populations (Chapman et al. 2021; Government of Fiji 2017; IPCC Working Group II, 2022; McLeod et al. 2019; Neef et al. 2018; Nelson et al. 2010; World Bank 2010).

According to the Sixth Assessment Report of the IPCC (IPCC Working Group II, 2022), the most climate-affected communities are those in urban informal settlements. In PICs, many informal settlements are situated in areas highly vulnerable to environmental hazards, such as low-lying, flood-prone riverbanks and coastal regions, alongside unstable slopes and near industrial or municipal waste sites (Gravelle and Mimura 2008; Phillips and Keen 2016; World Bank 2010). Consequently, these communities are exposed to frequent coastal and riverine flooding, extreme weather events, soil erosion, landslides, sea-level rise, and water contamination (Connell 2011; European Academies Science Advisory Council 2013; IPCC Working Group II 2014, 2022; Lata and Nunn 2011; UN-Habitat 2012a; World Health Organisation 2016). The rapid urbanisation in these regions has not only expanded urban areas, but has also led to an increase in the number and density of urban informal settlements, driven by a lack of access to adequate housing and shortcomings in urban planning (Connell 2011; International Organisation for Migration 2020; Kuruppu and Liverman 2011; Phillips and Keen 2016; United Nations Human Settlements Programme [UN-Habitat] 2012a, b).

Broader societal dynamics play a pivotal role in shaping how individuals and communities respond to climate threats (Aldrich 2015; Aldrich and Meyer 2015; Cutter et al. 2008; Hallegatte and Hallegatte 2016; Norris et al. 2008; Ungar et al. 2013). The availability of assets, access to essential resources, social capital and institutional support can determine a community's ability to sustain itself, adapt, or bounce back from challenges (Chaskin et al. 2001; Gaillard 2010; Goodman et al. 1998; Kretzmann and McNight 1993; Magis 2010). Climate adaptation strategies range from efforts to control and decrease the likelihood of climate-related issues, to coping mechanisms that reduce vulnerability, and avoidance tactics that mitigate potential impacts (Few et al. 2007). In the case of urban informal settlements, social marginalisation, lack of formal recognition in policy-making, and limited access to resources are real and present challenges (Adger and Kelly 1999; Blaikie et al. 1994; Carrasco and Dangol 2019; Shatkin 2004; Wigle 2014). Despite facing political, environmental, and socio-economic challenges, residents living in informal urban settlements are seen to demonstrate dynamism and resourcefulness (Carrasco and Dangol 2019; United Nations Economic and social Commission for Asia and the Pacific [UNESCAP] 2008) and invest in and self-organise their networks to implement adaptation strategies (Carrasco and Dangol 2019; Ortiz and Zarate 2004). However, the vulnerability of these communities is further exacerbated by the increased recognition that the distribution of national adaptation funds in low to middle income countries, such as the Global South, may fail to address the challenges experienced by the most affected communities resulting in increased calls for more equitable community-led strategies (McNamara 2013; McNamara et al. 2020; Sabates-Wheeler et al. 2008).

While challenges exist with terms such as Global South and Global North due to its reductionist view of social, economic, and developmental histories (Dados and Connell 2012), its inaccuracy in reflecting geopolitical boundaries (Horner 2020), and its misrepresentation of the geographical positions of Australia and New Zealand, the term persists in categorising low- and middle-income countries (generally situated geographically south) and industrialised, higher-income countries (primarily north of the equator). Although these terms reinforce Western and Eurocentric epistemologies (Connell 2007, 2014; Kothari and Cooke 2001), their use in this paper is intended to situate knowledge within existing framework through the use of existing terminology.

Community-based adaptation (CBA) emerges as a key strategy in this context (McNamara 2013; McNamara et al. 2020). Characterised by its focus on local-level engagement in climate-vulnerable areas, CBA employs participatory methods with local stakeholders to devise adaptation strategies that integrate cultural norms and local developmental needs to address underlying vulnerabilities (Ayers and Forsyth 2009). This approach emphasises the need for communities to both design and execute their adaptation measures (Dodman and Mitlin 2013; McNamara et al. 2020; Reid 2016). While foundational work on community-based climate change adaptation in the Pacific has been significant, these initiatives are relatively nascent, leaving the full extent of their community-level impacts yet to be ascertained (McNamara 2013; McNamara et al. 2020). Though these initiatives are designed to strengthen adaptive capacity by aligning with 'local realities in culturally appropriate ways', they face scrutiny for potentially harbouring paternalistic tendencies in their engagement methods (Ayers and Forsyth 2009; McNamara et al. 2020; Westoby et al. 2019). The imposition of Western-centric perspectives in adaptation efforts risks perpetuating top-down methodologies, and technical responses which facilitate predefined outcomes, which may undermine genuine community ownership and the effectiveness of such projects (Ayres and Dodman 2010; McNamara 2013; McNamara et al. 2020). This critique is compounded by the tendency of Western institutions to dictate adaptation strategies through technical and expert language, which can sideline local perspectives and obscure the cultural meanings tied to key adaptation concepts such as vulnerability, resilience, and risk (Bloemertz et al. 2012; Chavez-Rodriguez and Klepp 2018; Wise et al. 2014; Wolf 2011). Consequently, dominant narratives and top-down approaches can reinforce power imbalances and fail to account for local knowledge systems and adaptation strategies, diminishing the relevance and efficacy of interventions (Chavez-Rodriguez and Klepp 2018; Lampis, et al. 2022; Nightingale, et al. 2020).

In acknowledging the limited effectiveness of top-down approaches, alongside the nascent stage of community-based adaptation impacts, there is an increasing advocacy for community-led climate change adaptation strategies (Chavez-Rodriguez and Klepp 2018; Hay and Mimura 2013; McNamara et al. 2020). This shift necessitates a reevaluation of the role of external institutions in facilitating rather than directing adaptation processes, ensuring that initiatives are grounded in the knowledge, needs, and aspirations of the communities they aim to support (McNamara et al. 2020). Achieving this paradigm shift necessitates an in-depth exploration of the lived experiences of individuals within urban informal settlements, alongside their prevailing climate adaptation strategies. The research aims to not only reinforce but also refine the application of community-based adaptation in the Pacific by leveraging a qualitative research methodology known as Photovoice. Photovoice is recognised for its cultural relevance as a research approach that amplifies Indigenous and marginalised voices (Carroll et al. 2018; Castleden et al. 2008; Lewis and Swoboda 2016; Mitchell et al. 2020). This study utilises this technique to uncover the key determinants shaping community-led adaptation efforts in PICs, thereby providing a solid foundation for more effective and responsive adaptation initiatives (McMillen et al. 2014; Neef et al. 2018).

## Methods and materials

The Photovoice method was employed to explore the insights of residents from urban informal settlements, whose experiences have historically been underrepresented in climate adaptation research (Falconer 2014; Government of Fiji 2017; Phillips and Keen 2016). This approach facilitated an examination of individual and community-based adaptation measures, residents' perceptions of their effectiveness, and the factors influencing their implementation. This section details the research setting, rationale for the chosen method, and the data collection and analysis processes.

### Research setting

With its heightened vulnerability to climate change, Fiji has been exposed to major climate disasters in recent years, specifically cyclones and floods (Neef et al. 2018). Fiji's susceptibility to flooding is shaped by climate-induced factors, including sea-level rise, tidal and storm inundations, and heightened coastal erosion (Mann et al. 2016). While recent data highlight significant economic losses from fluvial and pluvial flooding (contributing to a loss in GDP 2.6% and 1.6% respectively), small-scale flood events are often underreported, impacting the accuracy of Fiji's overall risk assessment (Chapman et al. 2021; Government of Fiji 2017). This underreporting may be commonplace in informal settlements due to their unrecognised status and potential mistrust of government bodies (Carrasco and Dangol 2019; Ezeh et al. 2017; Satterthwaite et al. 2012, 2020). Human-induced climate events disproportionately affect informal settlements situated in flood-prone areas, where less permanent structures amplify the loss and damage during extreme weather events (Amuzu 2018; De Risi et al. 2013; Government of Fiji 2016; Phillips and Keen 2016; Shaheen 2018).

A major driver of this vulnerability is its rapid rate of rural to urban migration over the past 50 years. Fiji has the highest rate of urbanisation in Melanesia, with the number of people living in cities estimated to increase from around 50% of the population to 61% by 2030 (Phillips and Keen 2016; UN-Habitat 2012a, b). The rapid rate of urban growth along with limited urban infrastructure and affordable housing in Fiji’s capital city have contributed to urban growth corridors spreading to marginal lands in the Greater Suva Urban Area (GSUA) (Phillips and Keen 2016). The GSUA, which encompasses Suva City and the towns of Lami, Nasinu and Nausori, is estimated to be home to 244,000 people, with numbers expected to increase (UN-Habitat 2012a). An estimated 28,000 people in Suva are living in informal settlements on marginal lands (Phillips and Keen 2016; UN-Habitat 2012a).

The growth of informal urban settlements is attributable to overall population growth, the decline of the sugar industry, expired agricultural leases and the perception of better education and employment opportunities in urban areas (Bryant-Tokalau 2012; Fiji Bureau of Statistics 2017; Phillips and Keen 2016; UN-Habitat 2012a). As settlements grow, more people live in close quarters in flood-prone locations. Along with a lack of formal planning and inadequate infrastructure, high population density in informal urban settlements increases the flood exposure of communities (Amuzu 2018; De Risi et al. 2013; Phillips and Keen 2016; Shaheen 2018). Because they fall outside of the laws and regulations governing land ownership and use, informal settlements remain unrecognised and have limited prioritisation in government policies (Carrasco and Dangol 2019; Satterthwaite et al. 2012). Moreover, limited resources reduce the government’s capacity to implement climate mitigation and adaptation strategies (Carrasco and Dangol 2019; Satterthwaite et al. 2012; Satterthwaite et al. 2020).

This qualitative research was conducted in the context of a broader research programme, Revitalising Informal Settlements and their Environments (RISE), which is conducting a randomised controlled trial to test the effectiveness of water-sensitive, nature-based sanitation solutions across 24 informal urban settlements in Indonesia and Fiji to improve the health and well-being of these communities (Brown et al. 2018; Leder et al. 2021; RISE 2019, 2021; Spasojevic 2021). Working at the intersection of health, environment, water and sanitation, the RISE programme is employing community co-designed, water-sensitive approaches to sanitation, flood management, and water drainage to reduce community exposure to faecal contamination and water-borne diseases (Brown et al. 2018; French et al. 2021; Leder et al. 2021; RISE 2019, 2021; Spasojevic 2021). All settlements had been actively involved in the RISE parent study since 2017, maintaining familiarity with the local Fijian teams through previous engagement in broader RISE programme activities. Settlements were selected based on their susceptibility to and experience of coastal or riverine flooding and variations in their vulnerability to flooding and climate hazards. All participating settlements were located on riverbeds or coastal areas and typically had a medium-to-high population density, ranging from 19 to 115 dwellings per settlement (Leder et al. 2021). As part of the RISE parent study, community leases were established for all participating settlements, although land ownership varied across the 10 communities, involving a mix of community leases on state land or traditional land-owning agreements on native land (Leder et al. 2021).

This research took place in 10 informal settlements within the Greater Suva Urban Area (GSUA), during the broader research programme’s randomised control trial. A total of 60 households were selected to participate in the study based on their flood exposure, of which 49 households across the 10 settlements agreed to participate, with a total of 42 households completing the study, resulting in a maximum of five households participating per settlement. Given that intergenerational living in Fiji is commonplace, all adult household members who wished to participate were included, with interview sizes ranging from 1 to 4 adults per household interview. Although children were present, they did not take part in the discussions. Participating residents varied in sex (male and female), age (22–80 years), and ethnicity (Indigenous Fijian and Indo-Fijian). Interviews were conducted in iTaukei, Fiji Hindi or English, depending on household preference.

This study was approved by Monash University (Project ID 9396) and Fiji National University (CHHREC ID 310.20 and 137.19).

### Method rationale

The international focus on the implications of climate change for developing countries and their urban informal settlements is mounting (Satterthwaite et al. 2012; Tyler and Moench 2012; UN-Habitat 2011). The impact and the ability to respond to and recover from flood events are influenced by the structure and adaptive capacity of the community and the accumulated knowledge from previous flood experiences (Amoako 2015; Farías 2011). Given the complexity of climate adaptation, it is critical that different perspectives are identified and integrated into research, policy, and practice (Jentsch 2004; Kotze and Dymitrow 2021). While seminal cross-cultural research has been conducted, there are limited resources aiding the integration of diverse perspectives in North–South research (Broesch et al. 2020; Turner et al. 2024), meaning that the inclusion of various conflicting perspectives can be challenging (Jentsch 2004; Kotze and Dymitrow 2021). Qualitative research aims to identify the deeper meaning behind attitudes and behaviours; however, outsider mistrust of researchers may hinder the understanding of residents’ experiences of floods (Corburn et al. 2020; Government of Fiji 2017; Phillips and Keen 2016; Rashid 2009; Sholihah and Shaojun 2018). To overcome these potential barriers, this study employed the Photovoice method, a qualitative research approach, which was led by local Fijian teams with existing community relationships.

Photovoice emphasises the value of insights derived from personal experiences (Wang 1999). The qualitative research method integrates photography with in-depth interviews, aiming to empower communities to share their stories and experiences (Castleden et al. 2008; Wang and Burris 1997). Photovoice helps researchers to understand communities’ experiences, identify priority areas, and adapt the discussion to the interviewee’s position. Through collaborative knowledge co-construction, Photovoice can serve as a platform to amplify community voices, raise awareness, and drive action at both community and policy levels (Wang and Burris 1997). It has gained popularity over the past decade in a wide range of fields, including health, education, and international development, and empowers participants to become active agents in the research (Falconer 2014; Hormel 2016; Sutton-Brown 2015). Photovoice asks participating communities to capture lived experiences related to a predetermined project topic (Wang and Burris 1997). The collected photographs are shared for individual or group discussions, forming a comprehensive discussion which is used to raise awareness or advocate for change (Wang and Burris 1997).

When conceptualised as a tool within decolonial practice, Photovoice can actively engage in dismantling historical and complex power dynamics (Cornell et al. 2019). In this context, Photovoice has gained recognition as a culturally appropriate research method for empowering Indigenous and marginalised voices (Carroll et al. 2018; Castleden et al. 2008; Lewis and Swoboda 2016; Mitchell et al. 2020). However, achieving genuine participatory engagement presents challenges, with 'participatory' initiatives inadvertently upholding existing power imbalances among different groups (White 1996). Concerns persist regarding Photovoice's participatory rigour and its ability to address power imbalances (Carlson et al. 2006; Castleden et al. 2008; Howarth et al. 2014; Nykiforuk et al. 2011; Seitz and Orsini 2022).

### Data collection and analysis

The empirical research unfolded amidst the global coronavirus pandemic, an unprecedented period characterised by nationwide lockdowns and a halt to international travel. This situation necessitated a creative adaptation of the Photovoice methodology. Traditionally, a participatory approach that empowers communities to steer the research was modified to incorporate semi-structured photography and interviews. The empirical research was conducted during the global coronavirus pandemic, a period marked by nationwide lockdowns and the cessation of international travel. This context necessitated an innovative adaptation of the Photovoice methodology. Traditionally, Photovoice is a participatory approach allowing communities to guide the research; however, it was modified here to incorporate semi-structured photography and interviews. The adaptation involved prescribing the focus of the Photovoice approach, asking participants to photograph their experiences of flooding, its impacts, and their flood protection measures. To facilitate household-level interviews in light of COVID-19 restrictions, participants were specifically instructed to photograph measures they had personally implemented. The interview guides were designed around these photographs, employing an ordering activity that required residents to rank the measures by perceived effectiveness. While the semi-structured interviews concentrated primarily on the flood protection measures captured in the photo ordering activity, a preliminary review of broader experiences and impacts was undertaken to allow for context-specific probing during each household interview. This adjustment was crucial to ensuring that residents' perspectives remained central to the research, while also adhering to COVID-19 safety guidelines. It allowed for the collection of insights with substantial theoretical depth despite the constraints imposed by the pandemic.

Prior to the study, the lead author spent time in each settlement, observing and engaging with communities and co-created the research approaches with Fijian research partners (Turner et al. 2024). To challenge Western knowledge hegemony and enable residents to recount their experiences in their own words and language, Fijian research partners, also referred to as Fijian teams, were recruited to lead the Photovoice study. This approach aimed to mitigate the historical, political, and economic impacts of European colonial rule and generate culturally specific, rich data. By involving research partners deeply embedded in the history and culture of the study setting, the research not only acknowledged the Fijian teams as experts, but also ensured that residents’ accounts and socio-ecological contexts were accurately interpreted, reducing the risk of undervaluing specific contributions or Western-centric misinterpretation. A lack of cultural and historical understanding of colonialism, both broadly and within specific research contexts, can perpetuate power imbalances, hindering community engagement and reinforcing Western-centric notions of universal knowledge and an extractable objective truth (Gone 2018; Kotze and Dymitrow 2021; Schmidt and Pröpper 2017; Turner et al. 2024; Windchief et al. 2017). This perceived universality has historically led to the dismissal of alternative perspectives and limited the effectiveness of North–South research (Adams et al. 2017; McNamara and Naepi 2018a, b). By placing Fijian teams at the forefront of the research, a more multidimensional approach was ensured, enhancing understanding and engagement with community narratives, reducing universalist perceptions, and facilitating more effective knowledge co-construction (Anthony-Stevens and Matsaw 2019; Turner et al. 2024).

The Fijian teams conducted a socialisation process over a 1-month period, during which they visited and engaged directly with households and community engagement committees in each settlement. The purpose of the study, selection process, and voluntary participation details were communicated both verbally and in writing. A total of 60 households were approached as part of this research. Written consent was obtained from 49 participating households. Data was collected over a 3-month period (October to December 2020), during which researchers regularly communicated and shared their reflections with one another to maintain the ethnographic principles of having a local presence, conducting prolonged community engagement and holistically understanding people and their lives (Altheide 1987).

Participating households were provided with mobile camera phones and trained in their use by the Fijian teams. Households were asked to take up to five photographs of how they protect themselves and their homes from flooding. These photographs could be of individual, household, or settlement-wide flood measures, as long as a member of the household had personally contributed to its implementation. Upon completion of the photography task, camera phones were physically collected from each household, and the photographs and videos were downloaded from each device. The Fijian team members then reviewed the content. In cases where residents provided more than five photographs or submitted multiple images of the same subject, the most in-focus or contextually comprehensive photographs were selected. The chosen photographs were printed and numbered to facilitate their use in semi-structured interviews and transcriptions. Team members subsequently revisited the participants' homes with these printed photographs to conduct the semi-structured interviews.

The Photovoice method was instrumental in enabling Fijian research partners to successfully lead the data collection for this study while allowing the lead researcher to maintain an active role throughout the research process. The use of Photovoice enabled residents (both adults and children) to capture relevant images while allowing the researchers to familiarise themselves with the environment, communities, and residents and deliver ongoing training and supervision. Visual content consisted of both photos and videos that portrayed various aspects of flooding and flood responses, including flood-prone areas, flooding events, resultant damage, flood protection measures, and re-enactments of flood responses. The Photovoice method enriched participants’ descriptions of flooding events and enabled interviewers to explore individual stories in greater depth. Having access to photographs and videos prior to the interviews enabled the interviewers to pose tailored questions to each household, deepening the exploration of relevant topics. The use of photographs and videos in the interviews enabled Fijian team members to gain a deeper understanding of residents’ experiences of floods, with a focus on the factors influencing their flood protection measures.

Semi-structured interview guides were designed to ensure open and fluid discussions, while maintaining theoretical rigour. The interviews began with a focus on the overall experience of floods, then gradually transitioned to specific flood protection measures and an exploration of their photographs and experiences. Questions centred on their experiences in implementing flood responses, the factors influencing or inhibiting these responses, and the perceived effectiveness of responses. The interview approach was based on a funnel technique, moving from general to specific questions (Morgan 1997). General flood-related questions were aimed at eliciting emotional, mental, and physical experiences and information about flood locations and durations. Specific questions about flood protection strategies were designed to delve into the rationale behind implementation, lessons learnt, parties involved, and influential factors. To guide this discussion, participants were asked to arrange their flood protection photographs in order of response effectiveness, starting with the most effective measures. Fijian teams then facilitated a deeper exploration of the response efficacy of these strategies and the factors influencing their implementation.

While the primary focus was on the individuals responsible for implementing each flood response, other family members were encouraged to contribute information. All interviews were audio recorded, with duration ranging from 30 min to 2.5 h. The semi-structured approach and well-established relationships between the Fijian team and the communities in this study ensured the effective implementation of the study. Interview recordings were transcribed by members of the bilingual Fijian team using a three-step back-translation approach to ensure thorough and accurate translation and comprehension (Takahashi 2022). To validate the transcription approach, 10% of the interview audio recordings were back translated and compared by the principal researcher, and differences were reconciled prior to rollout across the remaining 90%. All transcripts were transcribed verbatim and translated into English. Ethnographic content analysis was employed to interpret the data (Altheide 1987). Transcripts were inductively coded using NVivo 12 to reveal flood experiences, flood protection measures, and influencing factors, including barriers and enablers of flood adaptation, leading to the development of an initial codebook (Charmaz 2006). Latent themes were identified to uncover underlying meaning and patterns within the codebook. These themes were reviewed with members of the Fijian research team and further refined based on cultural and contextual meanings (Altheide 1987; Charmaz 2006). Each theme was then analysed in detail. This approach enabled an identification of the theoretical concepts and narratives, the connections between concepts, the influencing factors, and the meanings attributed by residents (Altheide 1987; Boyatzis 1998; Crabtree and Miller 1999).

## Results

### Community perspectives on flood drivers and impacts

The use of Photovoice provided valuable insights into residents’ experiences with flooding and their commitment to flood protection. Through this method, adults and children documented over 600 photographs and videos across 10 settlements, capturing flood-prone areas, damage, protective actions, and even re-enactments of flood responses. These visual narratives offered a deeper understanding of flood impacts and strengthened connections between researchers and the community. By allowing participants to express their perspectives through imagery and narrative, Photovoice revealed systemic, societal, and individual influences with nuanced, context-specific details that traditional research might miss. Community engagement uncovered the factors influencing flood vulnerability, exposure, and protective strategies. This approach ensured that all community members were central to the research, identifying both barriers and enablers to adaptation and providing comprehensive insights into local strategies. Community voices are crucial in flood adaptation as they bring local knowledge, cultural context, and practical insights to the forefront, enabling the development of effective, sustainable, and equitable strategies tailored to the unique challenges faced by each community.

**Fig. 1:**
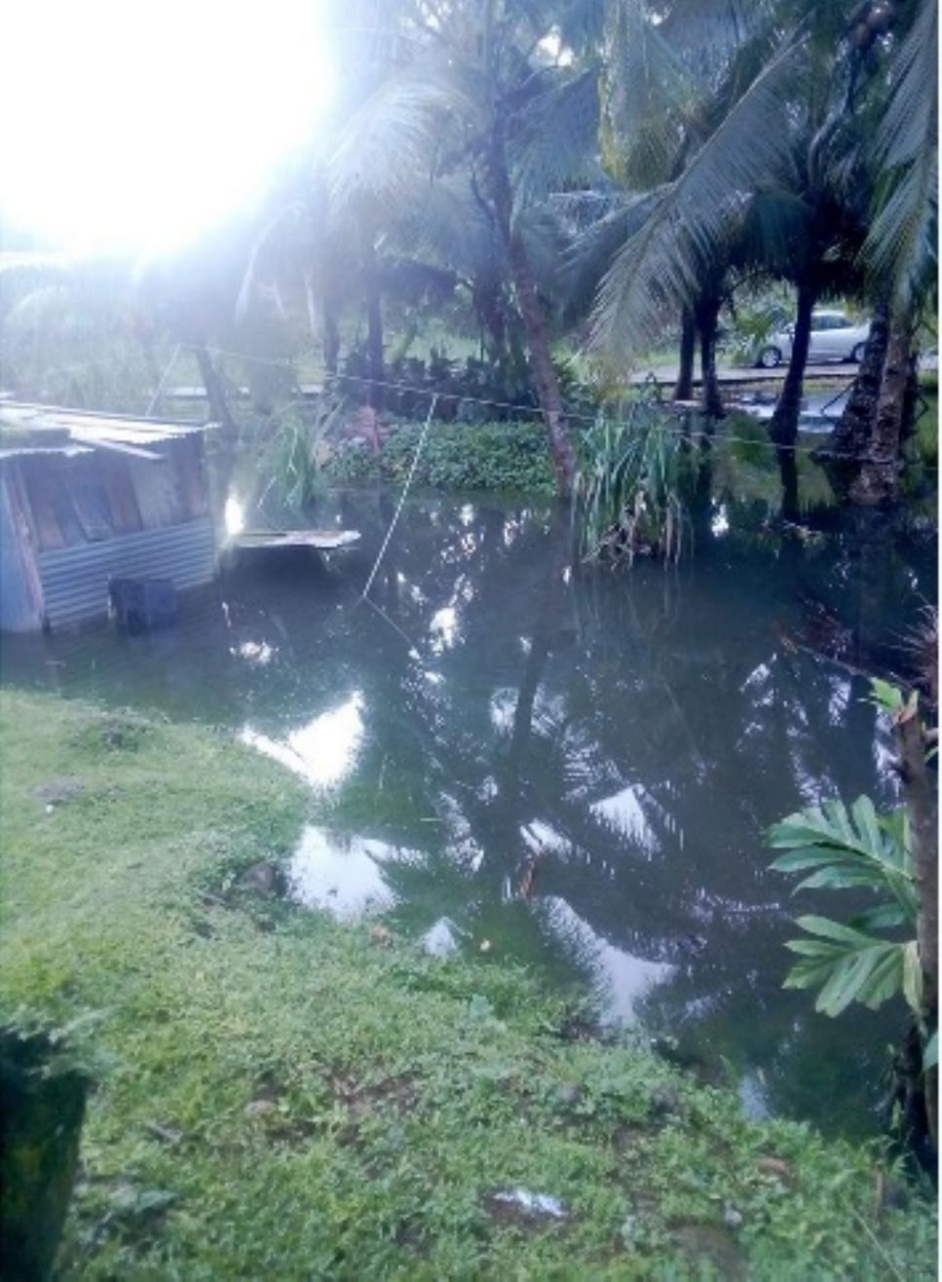
House submerged in floodwater

The significance of local perspectives is underscored by findings revealing the widespread impacts of climate-induced floods across all 10 participating settlements. The monthly king tide emerged as the primary cause of flooding, with floodwaters often taking 3–4 days to recede. Floods were more intense during heavy rain and cyclone seasons with residents recounting stories of their houses being completely submerged by floodwaters (see Fig. 1) and their belongings being irreparably damaged, along with harrowing accounts of children being swept away by floodwater:

*There was a time during a flood, I had sent my grandchild to cross the water. I remembered he crossed and almost lost his life on that particular day, but there were some people who got a hold of him and stopped him from being washed away by the strong current on that day.*

Quote ID: Settlement 7

**Fig. 2:**
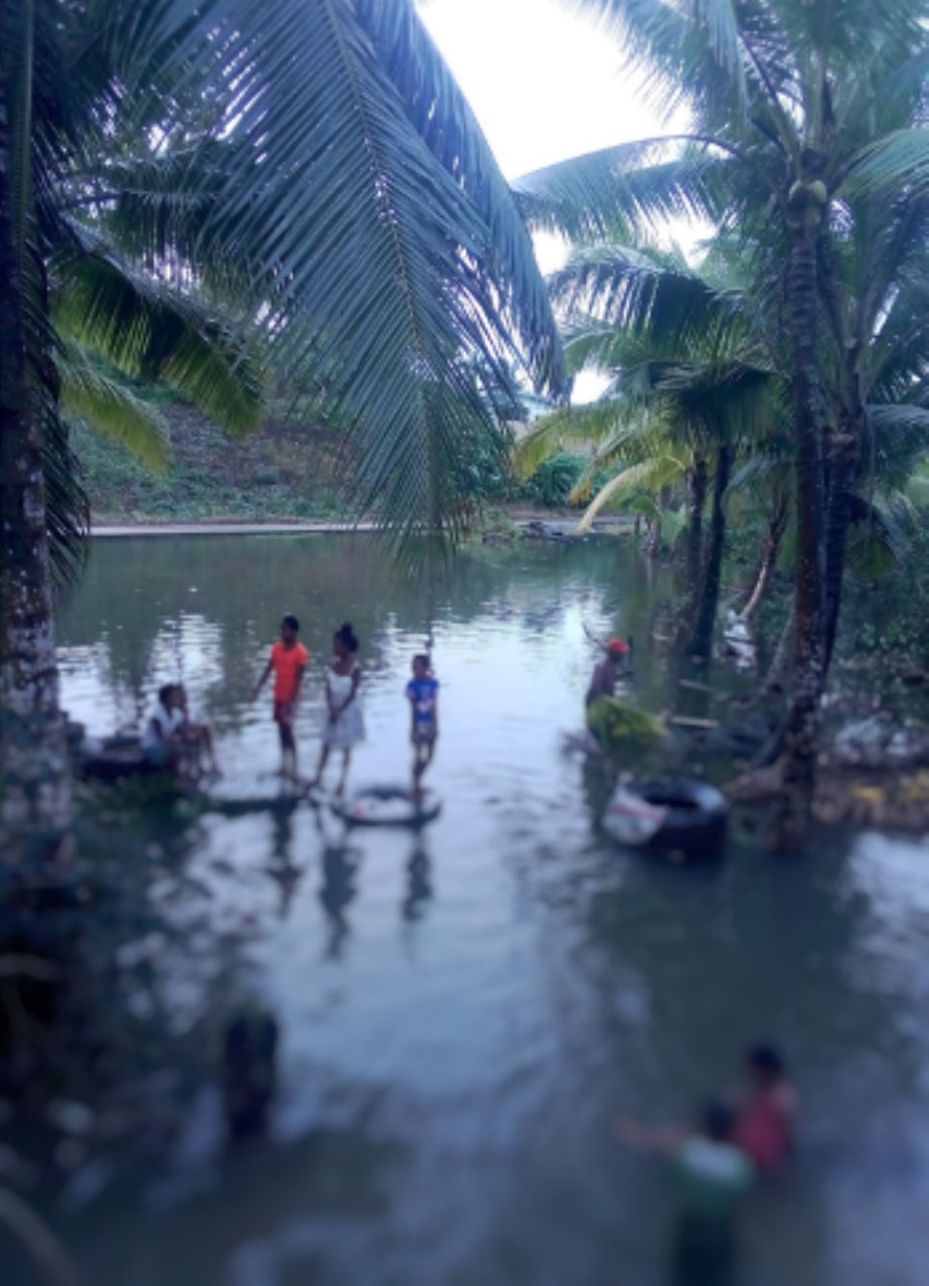
Children playing in floodwater

**Fig. 3:**
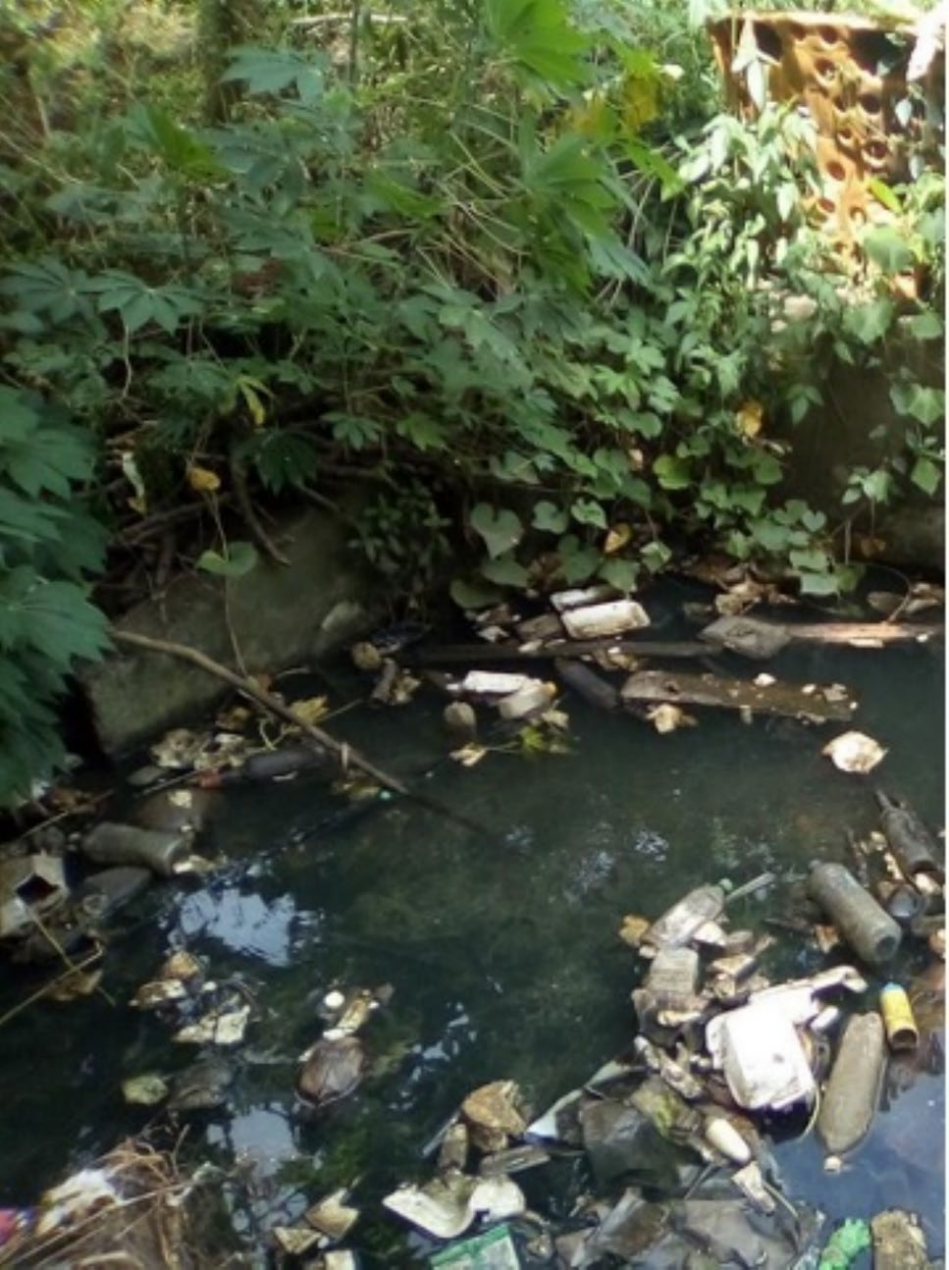
Rubbish and debris in floodwater

Direct and indirect experiences of flooding played a crucial role in their flood protection measures. The most critical driver of flood protection measures was the need to protect families and ensure the well-being of the community. Residents voiced health and safety concerns related to disease from contaminated floodwaters and injury from debris brought by floodwaters. The safety and well-being of children were particularly prominent, with residents expressing fear of children being swept away by floodwaters (see Fig. 2), being injured by glass and debris (see Fig. 3), and being susceptible to diseases such as scabies, asthma, and diarrhoea:

*When a flood occurs we feel really bad because water from the drain and the septic tanks flows in the flood. We want the drainage to be fixed because if the drain is fixed then the water and rubbish won’t be blocked, and we have small kids who can get sick easily. When flooding happens it’s very hard for us to go outside because of floods [lady washing dishes], [children making noise]…When there is flood we get dirty water in the tap and there is no water in the toilet system [and] it gets blocked. We always boil the water and give it to the children.*

Quote ID: Settlement 1

*During a flood we just stay indoors. We always wait for the water to recede before we leave. Reason being because of the diseases that the children are prone to such as scabies. For instance here there is also a high prevalence of asthma.*

Quote ID: Settlement 1

Exposure to pathogens, parasites, and disease vectors during flood events and their adverse health outcomes have been widely documented in the literature (Bain et al. 2014; Black et al. 2003; Ezeh et al. 2017; Harpham and Stephens 1991; Landrigan et al. 2015; Leder et al. 2021; Meijer et al. 2012; Nakagiri et al. 2016; Sverdlik 2011; van Ham et al. 2012). In line with the existing research (Borg et al 2021; Leder et al. 2021; Marfai et al. 2015; Okaka and Odhiambo 2019), residents attributed the spread of disease to poor house construction and settlement sanitation, the lack of drainage, and the activities of upstream communities, such as the dumping of rubbish and sewage in waterways. This adversely affected flood protection measures by blocking drains and exposing households to water contaminated with faecal material. Flood protection measures were seen as critical to ensuring the well-being and safety of families:

*I’ve seen that most of the houses here are located on the hill and do not really take notice of our implementations. We ourselves had implemented this because we are located directly where water flows across a wide path. That is why we have implemented those things, including the use of coconut husks and sand bags.*

Quote ID: Settlement 9

*I believe that this was going to happen. Whatever we do, there are consequences or challenges we face, but the truth is that the improvement of the wellbeing of the community, this enhances their preparedness to climate change.*

Quote ID: Settlement 6

Flooding was also perceived to affect residents’ emotional and economic health. Unlike findings from European flood protection research, the participants in this study had limited security, support, and infrastructure, rendering them more vulnerable to climate-induced flooding (Basel et al. 2020; Carman and Zint 2020; Dangol and Carrasco 2019). The burden of flood damage, loss of possessions, and the subsequent restrictions to employment opportunities fell entirely on the residents (Carrasco and Dangol 2019; Dangol and Carrasco 2019). The extent of these impacts was influenced by residents’ proximity to water bodies, and the effectiveness of their flood protection measures determined the impacts of flooding events. The compounded challenges undermined residents' financial capacity, placing them at a greater disadvantage and contributing to emotional distress and diminished optimism about their living conditions and future prospects (Carroll et al. 2010; Nöthling et al. 2024).

*Even though we live in a place of job opportunities for my children, when it floods it destroys our house items...Most of our items were destroyed by water. We thought that COURTS [the supplier from which they had purchased their household items] would give some assistance, but they didn’t...we just kept paying the instalments until it finished...Sometimes we thought of moving, and it is difficult because we already have our electricity and water, and it is difficult to move somewhere else. And it’s like our children have been cursed.*

Quote ID: Settlement 7

Despite these experiences, residents did not perceive settlement locations as unfavourable. Their proximity to urban centres, natural surroundings, and water resources enabled communities to preserve their social structure (akin to a traditional village) and subsistence lifestyles, while having access to educational and employment opportunities:

*We live in a good location. It’s easier for us, and we have adapted. It is near to town. If we want to eat fish, there is a place where we can fish. The small fish we get back from here. That is why my children have gotten used to staying here. It’s easier.*

Quote ID: Settlement 7

While flooding was acknowledged as an inherent aspect of community life, this acceptance should not be misconstrued as apathy (Carrasco and Dangol 2019). Flood protection measures were seen as crucial in ensuring the well-being and safety of kin. However, community connections and financial constraints restricted residents from moving away from their homes or settlements, implying that residents weighed up the costs (money and time) against the benefits (kinship, education and employment) of their living arrangements.

### Flood protection in informal urban settlements in Fiji

PICs are highly vulnerable to the effects of climate change and \often represented as lacking adaptive capacity because of the limited resources available to implement large-scale adaptation strategies (Mora et al. 2013; Pelling and Uitto 2001). However, these descriptions discount the local and traditional knowledge that has been attained by living with and adapting to environmental fluctuations and climate disasters for over a millennium (Barnett and Campbell 2010; McLeod et al. 2019; McMillen et al. 2014). The findings from this study highlight residents’ commitment to flood adaptation, with all 42 participating households implementing flood protection measures. In total, 240 flood protection measures were employed across the 10 settlements. Similar measures were grouped, generating a total of 32 flood protection measures (see Table 1).

**Table 1:** Flood protection measures

Flood protection measures	Implementation total
Categorisation	
Pre-flood warning signs and observations	
Tracking weather patterns and events (e.g. dark clouds or full moon)	4
Keeping track of water levels against environmental or spatial flood markers (e.g. tree trunks or house posts to see where the water is in relation to their home) and sharing information with other members of the community	8
Observation of flood-prone areas	2
Preparing and safeguarding pre-flood	
Stocking up on essential items such as groceries, water, candles, fuel, and medicine	8
Using communal bathroom facilities located in low-lying areas outside the property pre-flood	1
Tying up items that might be swept away	3
Placing possessions in higher locations (e.g. electrical items on high shelves or top level of the house)	14
Placing sandbags, freestanding corrugated sheets, and pallets next to the house to control and redirect the water	7
Collecting and clearing rubbish and debris from the compound and drains pre-floods to prevent a buildup in drains and culverts	11
Retreating or relocating during floods	
Staying indoors during a flood and keeping children at home during floods (staying home from school)	15
Moving up to the second floor of the house for those with a second storey	2
Moving to higher ground or evacuation centres (including livestock)	11
Utilising, borrowing, or paying for a boat as a mode of transport during a flood (e.g. transporting children to school and adults to employment)	6
Using Styrofoam trays to carry possessions across floodwaters	1
Lessening the impact during flood	
Clearing rubbish and stirring water during a flood to prevent mud from being left behind	2
Recovering from flood impacts of flood	
Washing, drying, and repairing water-affected items such as clothes and furniture in the sun	8
Cleaning up rubbish, debris, and floodwaters in and outside the home post-flood	25
Collecting anything swept away by floods (e.g. tyres, small wooden planks used as bridges)	1
Unblocking and clearing mud, debris, or rubbish from existing drains, culverts, and watercourses post-floods	20
Adapting the built environment pre- and post-flood	
Widening or deepening existing drains and culverts to increase the flow of floodwater	5
Arranging and memorising the location of tyres, stones, and wooden planks in flood-prone areas to create a pathway during floods	15
Placing tyres around house posts to brace the house during floods and reduce water damage to wooden posts	4
Digging new drains or moats around the house or compound	2
Raising the height of the house on wooden stilts or concrete blocks (including livestock coops)	15
Building flood walls or barriers (e.g. tyres, stones, wooden logs, or coconut husk fences)	10
Raising the ground level of the compound or low-lying areas of the settlement by adding soil, sand, wood, debris, iron roof sheets, and stones	10
Relocating the entire house to higher ground	2
Burying stones, iron roof sheets, tyres, wood, and coconut husks in soil-filled areas to hold soil and prevent erosion and landslides	8
Adapting the natural environment pre- and post-flood	
Removal of trees, long grass, and flowers from the drains and waterways to increase the flow of water and prevent rubbish and debris from becoming caught	4
Planting grass, taro, flowers, and coconut or sandalwood trees around the house and compound to slow the flow of floodwater and prevent soil erosion	15
Replanting and restoration of coastal mangroves as natural flood barriers	1

Despite the terminological debates and conceptual fuzziness (Manyena et al. 2019; Smit and Wandel 2006), climate adaptation is generally understood as the method through which adjustments are made in response to the direct or anticipated impacts of climate change. This process aims to minimise or prevent negative outcomes while also seizing any beneficial opportunities that arise within human systems as a result of these climatic changes (Mach et al. 2014). Adaptation strategies in this study’s context were defined as incremental changes, modifications, or adjustments designed to lessen flood impact, which can be performed before, during, or after flood events (Béné et al. 2014; Manyena et al. 2019).

The flood protection measures identified in this study were designed to prevent or mitigate residents’ exposure to floods; however, their intended outcomes differed. Observation of weather patterns and water levels was done to mitigate flood risk and help in decision-making and the implementation of flood protection measures. The placement of tyres and wooden planks enabled residents to move freely during floods while limiting their exposure to floodwaters. The clearing of drains reduced the likelihood of floodwaters breaching drain banks and inundating residents’ compounds. Raising houses on stilts or blocks was done to prevent contaminated floodwater entering homes. Residents implemented an average of five to six flood protection measures to mitigate or avoid flood impacts. The choice of flood protection measures was influenced by a number of factors which will be elaborated on in the subsequent section.

### Factors influencing flood protection measures in informal urban settlements

#### Local environmental knowledge

Flood measures were informed by local place-based knowledge of the climate, weather patterns, and environmental markers. Flood measures differed according to when they were implemented in the flood timeline and the speed and intensity of environmental changes. For example, if residents considered floodwater levels to be of minimal risk, they simply stayed indoors and placed their possessions on higher shelves. If the floodwater level was perceived to exceed safe limits, residents evacuated to higher ground. Weather patterns were used to identify impending flood risk, while historic knowledge of flood patterns was used to determine changes to community exposure and influence flood responses:

*One thing I saw the most was the change in the increased amount of flooding, and we’ve been living here for a long time. Before, most of these houses were just sitting on the ground...Hence my decision to raise my house. You’ll notice that most of the houses here have posts. The houses are on posts because of the flood. But when we experience flooding the whole community floods.*

Quote ID: Settlement 1

*Umm because the water—my village is right next to the river. Whenever I see dark clouds, I know straight away that there’s going to be rain, and so I tell them to get ready while it’s still daytime.*

Quote ID: Settlement 2

**Fig. 4:**
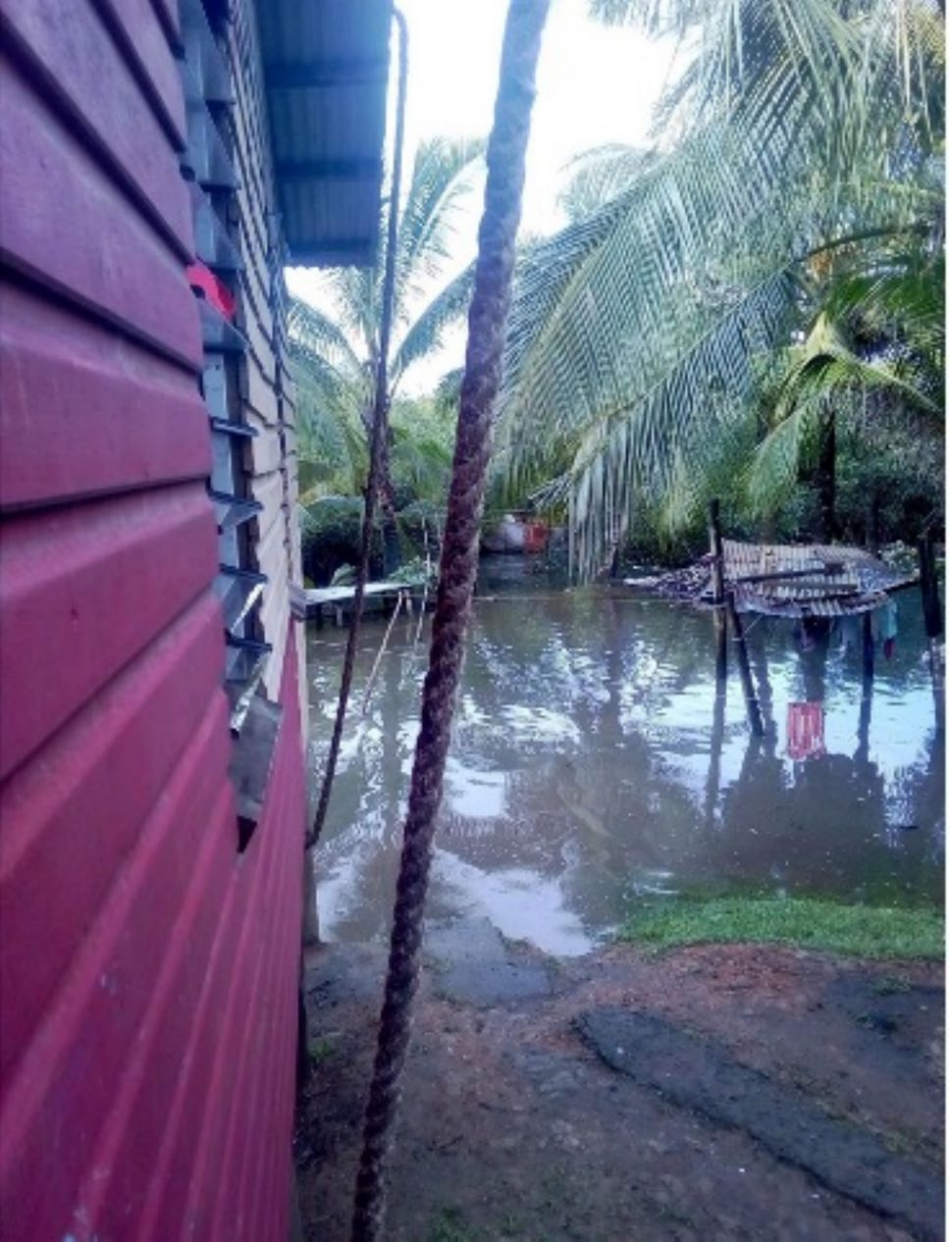
Regularly checking the location of water in relation to the home

Residents emphasised the importance of observing water levels and heeding warnings when flood events exceeded local environmental boundaries (see Fig. 4).

*Preparing ourselves to move with essentials to take especially food so that by the time water tends to come into the community everyone is well prepared and have moved out of the area not forgetting the children.*

Quote ID: Settlement 4

The intensity of flood ingress determined whether residents stayed inside and secured items or evacuated to higher ground. Residents’ physical ability to act determined whether they sought or received assistance from members of the community, such as youth or neighbouring families. During high-risk events, leadership played a crucial role in evacuation. Settlement leaders such as village or family heads, or religious leaders communicated flood risks and galvanised members of the community to evacuate. Historic knowledge and residents’ understanding of the local environment played a vital role in determining risks and implementing flood protection measures (Lebel 2012).

*It’s like the first thing we do—we’re not careless or sleep when there’s a flood warning; we always look out when there’s a rise in water level. It’s good we prepare everything in the day compared to night. When there’s a warning, our relatives that stay at the top usually tell us to start moving up in the day because by night accidents do happen, and in the day we can see what we’re doing.*

Quote ID: Settlement 5

*The first thing I think of is, if I do not take any initiative, there won’t be anyone else that will come and help us in the problem we face. That is why I am always prepared. I always tell them if the rain falls, we always place the roofing iron in front. If it rains more heavily, for those to stand prepared, because we never know where water would flow out from because of the force of water that flows down this place, and the first thing is it gathers at the bottom of the house because everything stays there.*

Quote ID: Settlement 1

#### Intergenerational knowledge and community cooperation

Knowledge of flood response measures was shared across the community and passed down by elders and leaders of common kinship groups. Children were taught about environmental markers that indicate flooding, such as points on certain trees, from an early age. Residents spoke of learning how to dig drainage channels in childhood and continuing to apply that knowledge years after their parents had passed. Intergenerational teaching included how to implement flood protection measures, the notion that residents should help their neighbours, and the benefits of working together to improve the well-being of the settlement. While these practices were ingrained in resident narratives, residents advised that the presence of different religious denominations and leaders in settlements, the influx of new residents with limited ties to the community, and the lack of direct community-wide benefits hindered the implementation of community-wide flood protection measures in most cases. This resulted in the majority of flood protection measures being carried out by kinship groups or smaller subsections of the community, and variance in community-wide participation across the settlements:

*There are many church denominations that live here, but we do not work together. It’s the Methodist denomination that is more dominant here. We know that it is us who carry out the responsibilities here. I have never seen a time where everyone came together to solve a problem.*

Quote ID: Settlement 8

Whole community or community-wide flood responses were less frequent and predominantly limited to settlement cleanups. While this activity was not always explicitly implemented for flood protection, communal activities were seen as beneficial to flood protection as they help reduce the amount of rubbish washed into waterways and drains:

*There’s no working together [on flood responses], just [community] clean-up campaign...because this place is not like a village. If it was a village, people would cooperate...if there could be a headman of this settlement to go around when there’s a heavy rain checking if everyone is okay and if there’s a family that needs help—we just need someone to oversee.*

Quote ID: Settlement 9

*The measures that we implemented as a community are cleaning the community together, the drain, umm, a lot of time we try and open the entrance for that area. Overcome that first, so that water flows well here. We help one another, and when we clean the community together it is to mitigate flooding.*

Quote ID: Settlement 7

A common theme across settlements was residents’ desire for whole of community or community-wide implementation of flood protection measures to reduce the impacts of flood exposure. According to residents, settlements with traditional village structures enabled stronger cooperation because a dedicated leader would check on residents, offer support, and ensure that the community worked together. Residents noted the importance of village heads and community, and religious and council leaders in encouraging the community to cooperate and implement community-wide flood responses. However, the study revealed a lack of clarity regarding the definition of a 'village', as evidenced by inconsistent descriptions provided by residents from the same community, suggesting further exploration of this concept is needed.

#### Low-cost measures were the most prominent

**Fig. 5:**
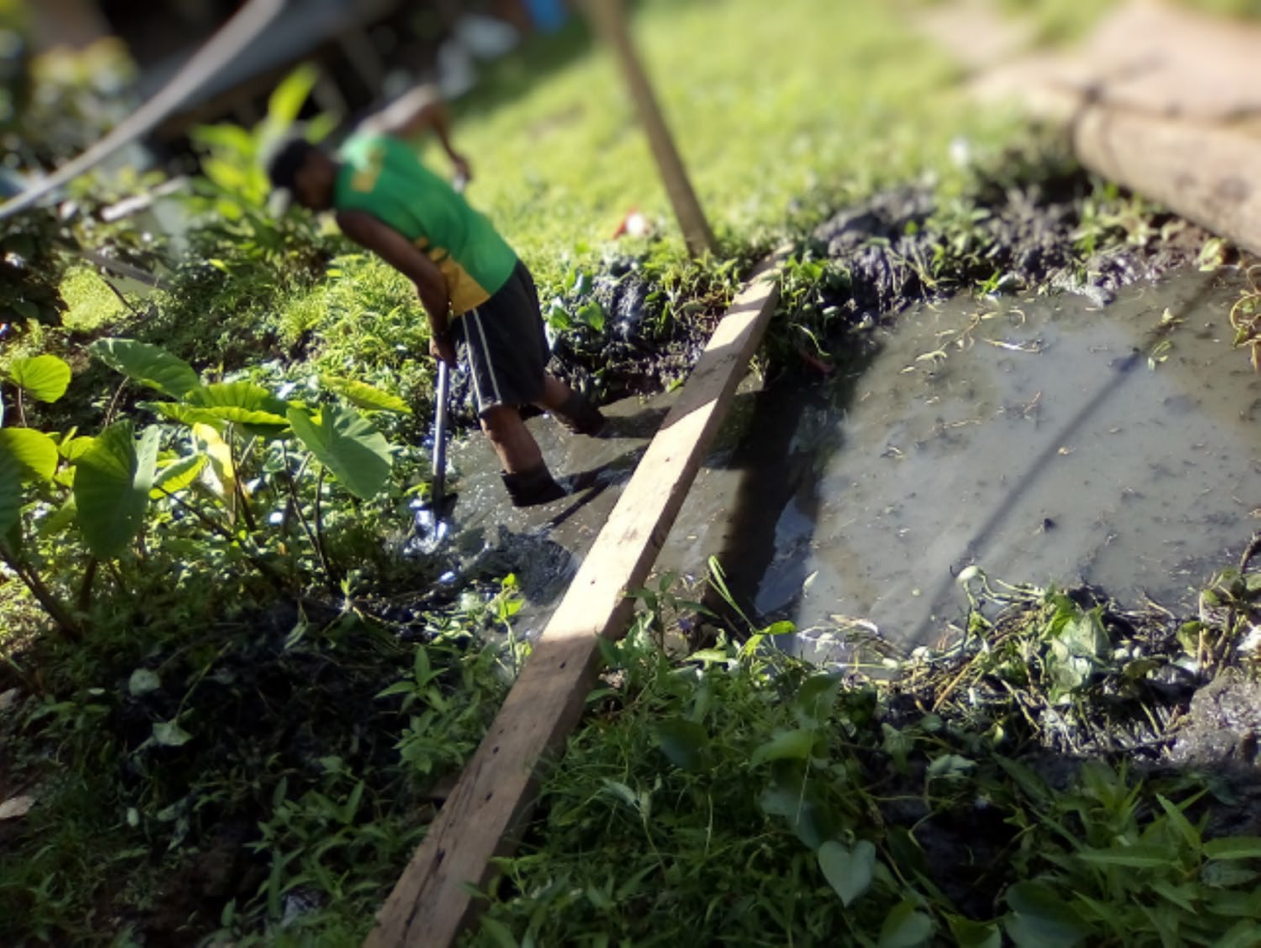
Mud, debris, and rubbish from existing drains, culverts, and watercourses

Consistent with existing flood literature, the most prominent flood protection measures were those that were low in cost and relied on physical labour for implementation (Marfai et al. 2015; Mulligan et al. 2017). The most common measures were the post-flood removal of water, rubbish, and debris from houses and the surrounding compound and the clearing of existing drains (see Fig. 5).

The findings indicate that physical or labour-intensive measures increase when financial resources, materials, and equipment are limited. The labour intensity of these measures was determined by residents’ ability to garner support and assistance from their kin or neighbours—a finding consistent with Marfai et al (2015). Similar to Islam, et al.’s 2018 findings, lower social capital and limited access to materials or equipment increased the physical labour required to implement flood protection measures:

*Because we live on our land, most of the work requires manpower, and there are no machines or for something to help us, just us, delegation of work, working together as a family, which makes us accomplish the task to help encounter the challenges that affect us.*

Quote ID: Settlement 3

*Some families here, when the tide rises, the water gathers in the drain. The drain that runs beside the house will see the water level rise, almost touching the bottom of the house. We always go and help out with the other man to clean up the drain.*

Quote ID: Settlement 6

**Fig. 6:**
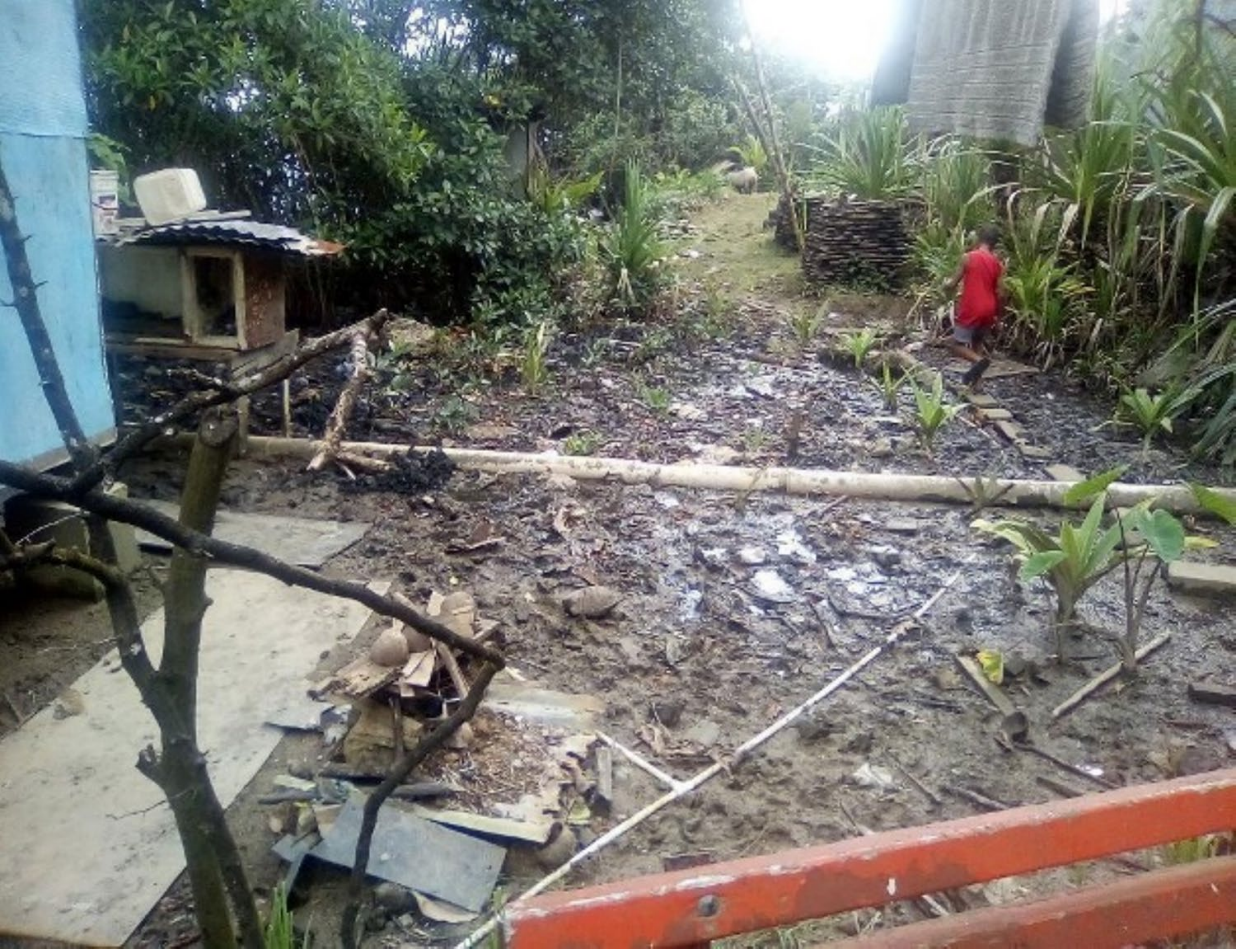
Pathway made from stones and wooden planks

**Fig. 7:**
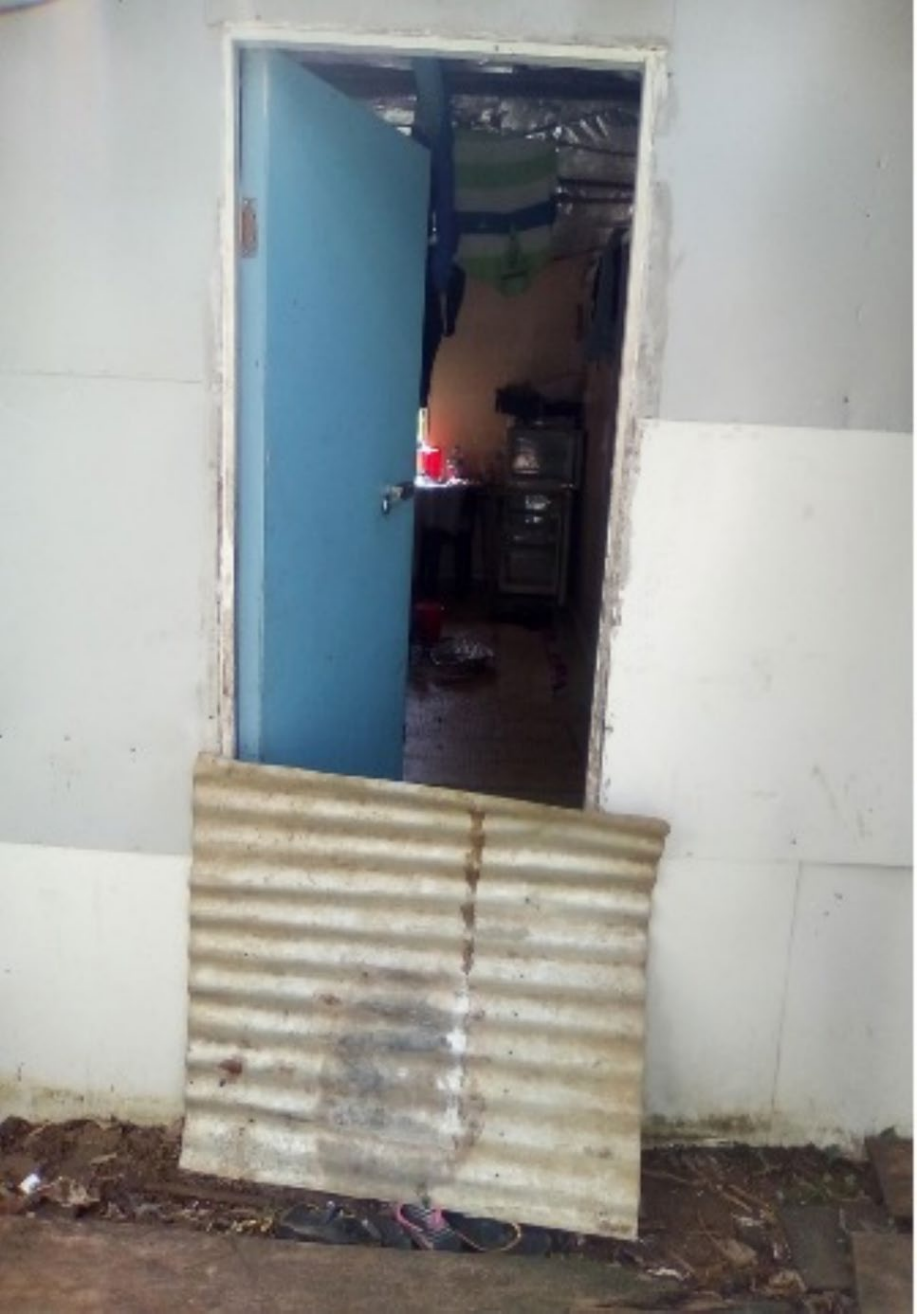
Corrugated sheets used to redirect water during floods

To reduce the cost of flood protection measures, residents commonly repurposed materials such as old tyres, iron roof sheets, soil, stones, and debris from trees (see Figs. 6 and 7).

Residents utilised these materials to redirect and control floodwaters and help them move freely during floods. Despite their frequent use, low-cost flood protection measures were typically only effective for a single flood event, and thus needed regular implementation. Similar to existing literature, the findings from this study indicate that the low response efficacy of these flood protection measures may perpetuate existing vulnerabilities and reduce residents’ flood resilience over time (Asian Development Bank 2019; Klijn et al. 2015).

### Modifications to prominent practices

Residents were seen to make incremental improvements to their flood protection measures over time. Participants learnt from their previous failures and modified their flood protection measures, which is consistent with the literature (Béné et al. 2014; Manyena et al. 2019).

*There were about 60 tyres when it did it in the first place. I also fill it with loose gravel to strengthen it. So it can be their walkway as well. Now the tyres are getting buried. But, for me, I continue to work on the changes. That is part of their current problem, because they never revisit what they’ve already done. I am thankful, although we may not always be on the same page. I will continue with this work till I go back to the village. Thank you very much.*

Quote ID: Settlement 2

They also spoke of knowledge being shared through kinship and community networks before and during the implementation of flood protection measures. The most common modifications were made to the most frequently used measures—the post-flood removal of water, rubbish, and debris and the clearing of existing drains. In these instances, residents changed the frequency and timing of measures (e.g. cleaning rubbish and debris before floods happened) or improved existing practices by making modifications to their implementations, (e.g. utilising soil to raise the ground height of the compound or planting vegetation on soil removed from drains).

*It [clearing the drains] has benefited a lot by implementing this response now the water is not blocked. By doing this now water is not blocked and the compound is a bit higher now [repurposing soil from drains to raise the ground level of the compound]. It is now better for children to go to school. By filling up the compound with soil water won’t be blocked and if the drain is big then less water will be in the compound and my family will not be affected by flood.*

Quote ID: Settlement 10

The aim of these modifications was to enhance the effectiveness of existing measures, but cost constraints limited the extent of the improvements:

*If we were able to improve we’d actually put some more blocks [laughter] along the sea wall, along the- where all the tyres are at the moment it be better if we had other blocks as well so that would one way we would help improve it and it would help the families as well not only our family but other families as well because given their situation most they cannot, aren’t able to purchase the cement blocks so they are making do with what they have.*

Quote ID: Settlement 4

**Fig. 8:**
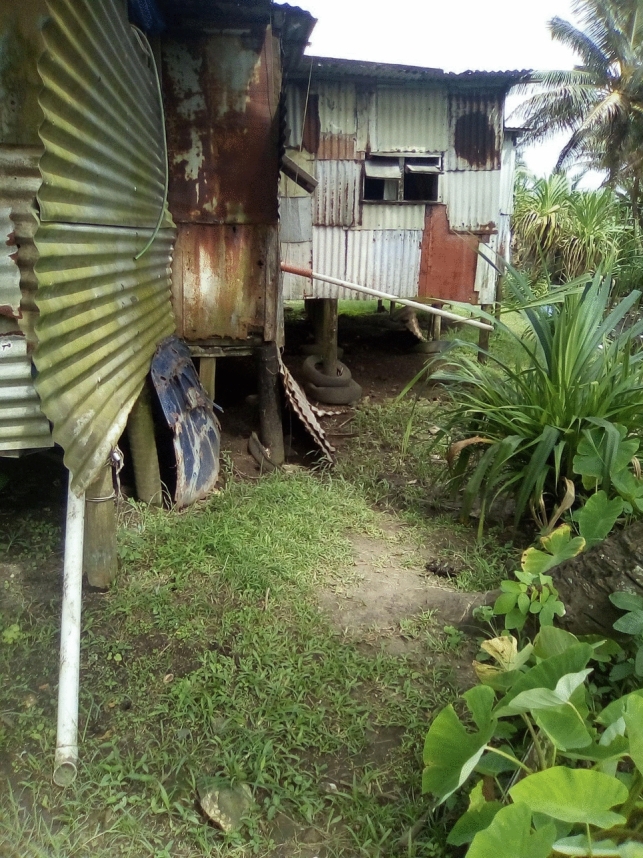
Taro planted to help with food at home

**Fig. 9:**
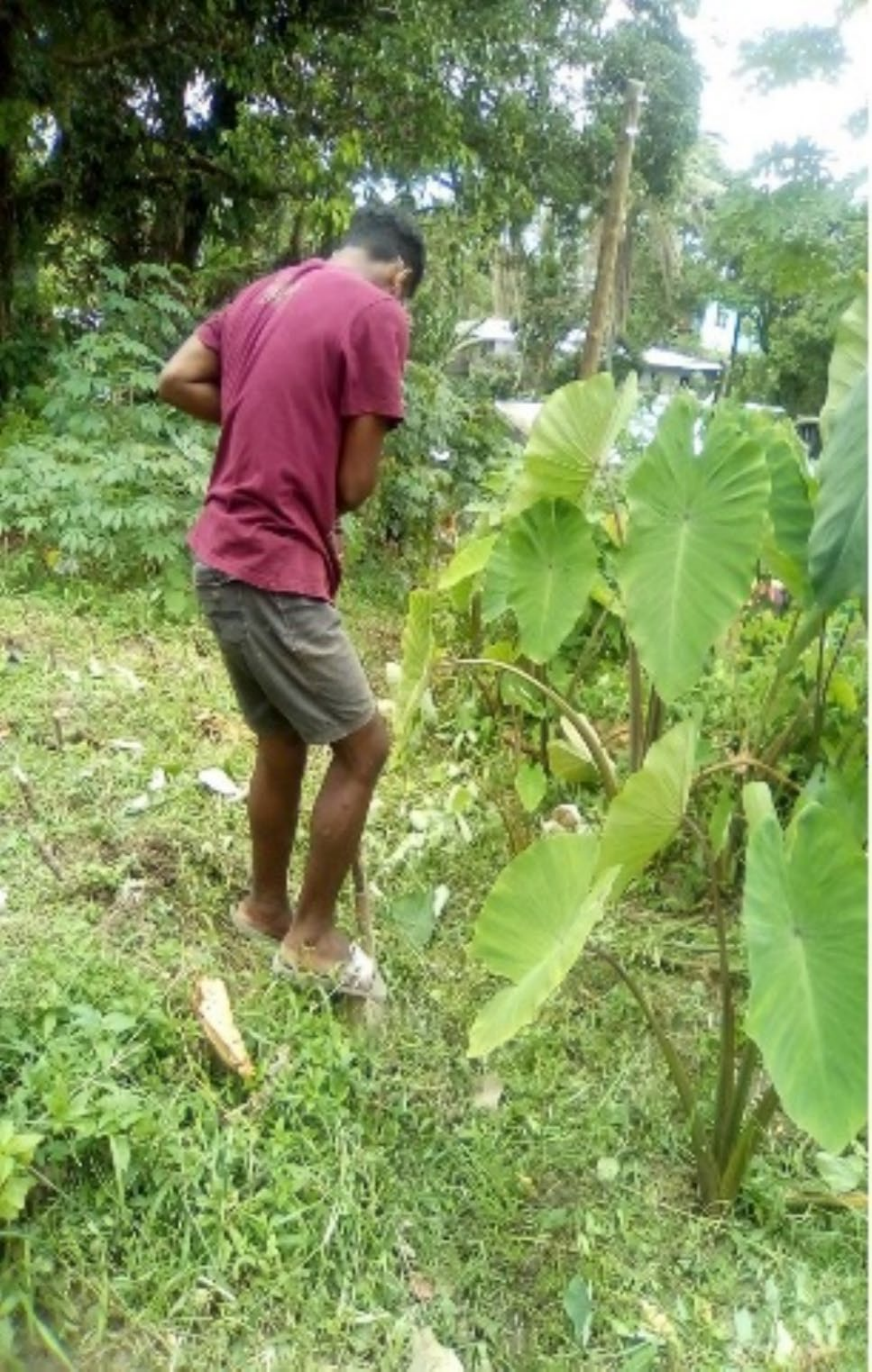
Growing grasses, taro, flowers, and trees around the house to slow floodwater, strengthen the soil, and reduce erosion

Residents favoured multipurpose flood responses such as nature-based solutions. The most common multipurpose measure was the planting of pandanus and coconut trees, flowers, grasses, and edibles such as taro, spinach, and chillies to prevent soil erosion and slow the flow of floodwater (see Figs. 8 and 9). These measures also helped to buffer the effects of floods by diversifying income streams or generating additional sources of food (e.g. weaving pandanus leaves into saleable items such as mats and baskets):

*The pandanus leaves, when it’s planted, also sucks the water that is flowing on the ground; when it is muddy it is strong enough to suck the water. When it is planted there—that is a muddy area—pandanus leaves are planted there and still are dry. At the back here is also muddy. It’s meant to be planted there and also at the edges of the water.*

Quote ID: Settlement 2

*Umm, umm [nodding], it’s planted because that is also an income for us at home. It also protects the soil and also for weaving the mats that we are sitting on [refers to photo]. These taro leaves are planted here; it helps us with food at home. This one is fenced because—the water area, that side is where the pandanus leaves are planted.*

Quote ID: Settlement 2

*These [flowers] are to help with the food at home. The chillies are placed on this side. These are the cabbages that were given by Habitat. They just helped out. These ones I just planted to help with the protection—that area is muddy, so what I did is put soil on the tyre, plant eggplant. The ones in the bucket, I’m planting cabbage in it to help with the food at home.*

Quote ID: Settlement 2

### Permanent structural flood responses were the most effective

**Fig. 10:**
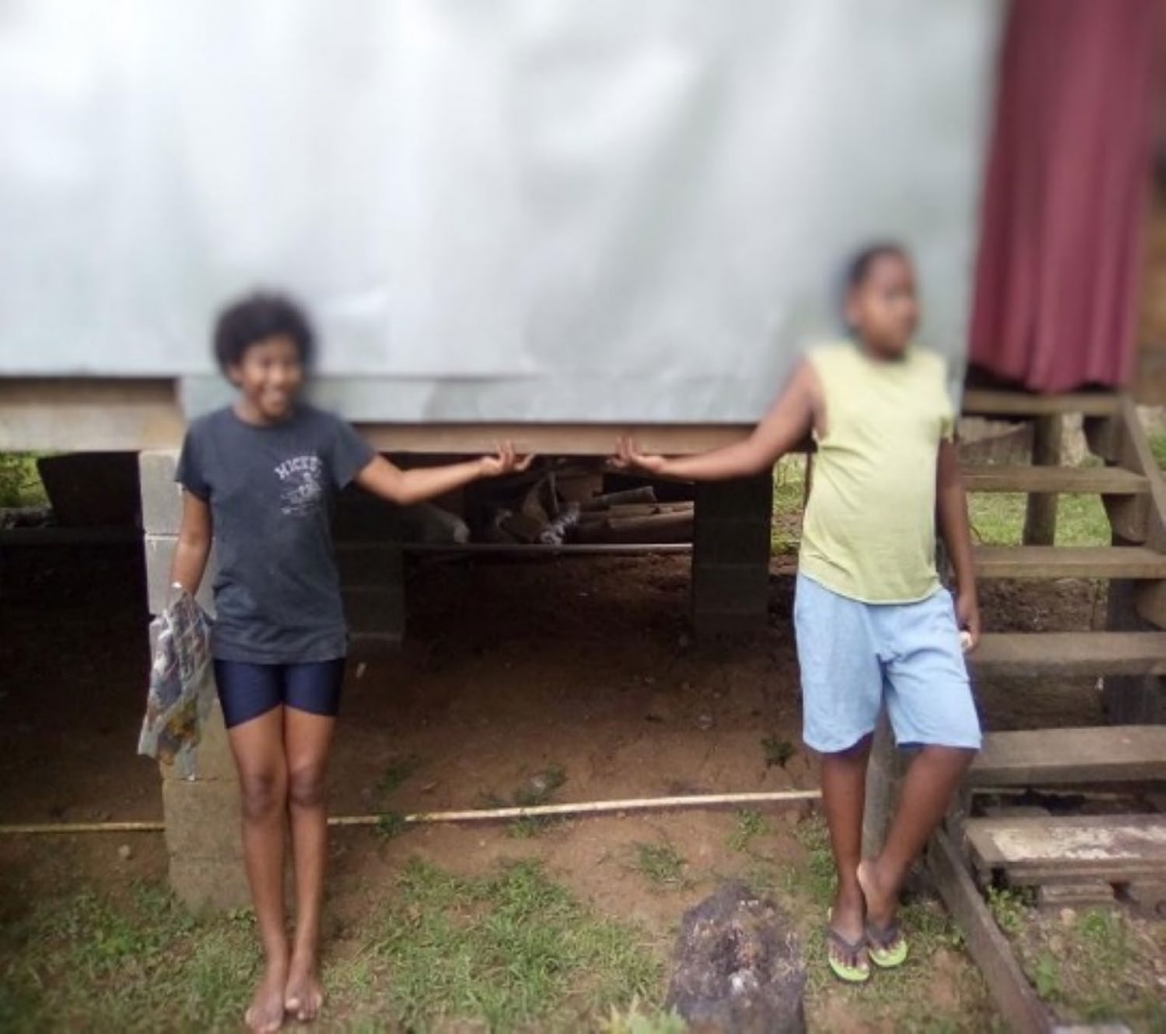
House raised on cement blocks

**Fig. 11:**
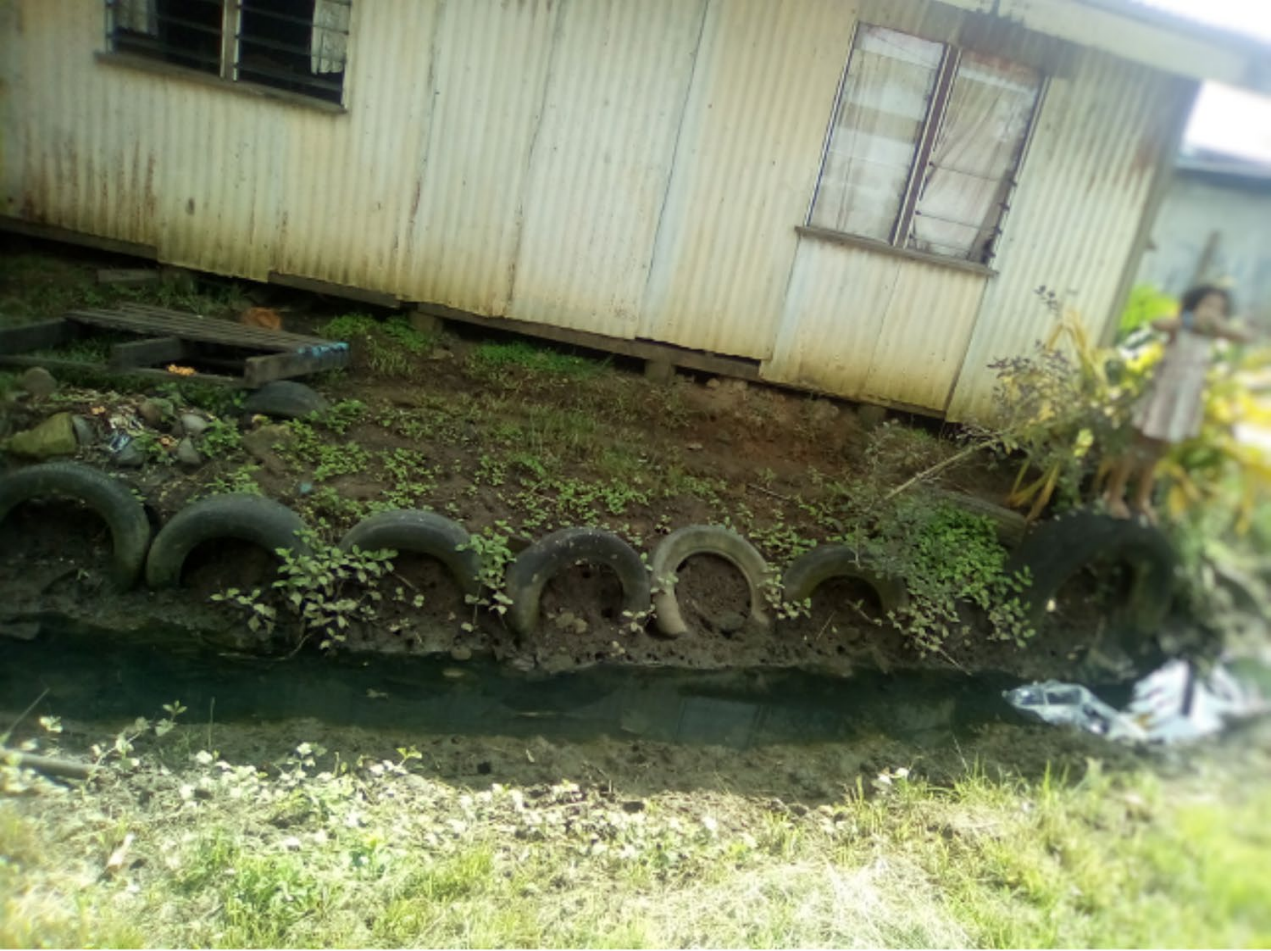
Permanent building up of soil underneath and around the house

Participants reported permanent structures as the most effective flood protection measures, because other measures would only be effective in stopping, slowing, or redirecting floodwater. Common structural measures included raising homes with wooden posts or concrete blocks, or raising the ground level with tyres, stones, iron roof sheets, and vegetation (see Figs. 10 and 11).

However, despite their effectiveness, these measures were costly and often required residents to use their savings or seek external loans. Financial limitations made permanent structural measures inaccessible for some residents:

*Not all the houses would be able to afford to pay for the preventative measures…*

*since we are living in this kind of area or settlements most are living with whatever they can afford or what they can make do with so those are the constraints and I think aah the conditions of their houses is another contributing factors is a constraint because that is what they can afford at the moment with their houses so if flooding waters goes in it’s what they can live with at the moment so probably money would help them build new houses with longer posts.*

Quote ID: Settlement 4

Financial limitations and a certain resignation to vulnerability emerged as recurring themes in residents' narratives, highlighting how economic disparities and the lack of proper housing solutions contribute to a continuous cycle of risk and recovery. This situation emphasises the need to tackle systemic obstacles that hinder communities' ability to respond to and recover from crises effectively. Notably, residents who had the resources to make structural improvements often experienced a diminished sense of fear and vulnerability to flooding, underscoring the significant impact that addressing these broader issues can have on enhancing community adaptation and resilience:

*Before a flood, because our house is on higher concrete posts, and so we do not worry about anything because the water does not reach the doorstep or the bottom of the house.*

Quote ID: Settlement 6

Despite being more costly, these measures still involved physical labour (see Table 2).

**Table 2:** Flood protection inputs and outcomes

Flood protection inputs and outcomes			
Categorisation	Cost	Labour	Permanence
Pre-flood warning signs and observations			
Tracking weather patterns and events (e.g. dark clouds or full moon)	N/A	Low	Low
Keeping track of water levels against environmental or spatial flood markers (e.g. tree trunks or house posts to see where the water is in relation to their home)	N/A	Low	Low
Observation of floodwater over time	N/A	Low	Low
Preparing and safeguarding pre-flood			
Stocking up on essential items such as groceries, water, candles, fuel, and medicine	Low	Low	Low
Using communal bathroom facilities located in low-lying areas outside the property pre-flood	N/A	Low	Low
Tying up items that might be swept away	N/A	Low	Low
Placing possessions in higher locations (e.g. electrical items on high shelves or top level of the house)	N/A	Low	Low
Placing sandbags or freestanding corrugated sheets next to the house to control and redirect the water	Low	Low	Low
Collecting and clearing rubbish and debris from the compound and drains pre-floods to prevent a buildup in drains and culverts	N/A	Medium	Low
Retreating or relocating during floods			
Staying indoors during a flood and keeping children at home during floods (staying home from school)	N/A	Low	N/A
Moving up to the second floor of the house for those with a second storey	N/A	Low	N/A
Moving to higher ground or evacuation centres (including livestock)	N/A	Medium	N/A
Utilising, borrowing, or paying for a boat as the mode of transport during a flood (e.g. transporting children to school and adults to employment)	Low	Medium	N/A
Using Styrofoam trays to carry possessions across floodwaters	Low	Medium	Low
Lessening the impact during flood			
Clearing rubbish and stirring water during a flood to prevent mud from being left behind	N/A	Medium	Low
Recovering from flood impacts of flood			
Washing, drying, and repairing water-affected items such as clothes and furniture in the sun	Low	Low	Low
Cleaning up rubbish, debris, and floodwaters in and outside the home post-flood	N/A	Medium	Low
Collecting anything swept away by floods (e.g. tyres, small wooden planks used as bridges)	N/A	Low	Low
Unblocking and clearing mud, debris, or rubbish from existing drains, culverts, and watercourses post-floods	N/A	Medium	Low
Adapting the built environment pre- and post-flood			
Widening or deepening existing drains and culverts to increase the flow of floodwater	N/A	High	Medium
Arranging and memorising the location of tyres, stones, and wooden planks in flood-prone areas to create a pathway during floods	Low–Medium	Medium	Medium
Placing tyres around house posts to brace the house during floods and reduce water damage to wooden posts	Low–Medium	Medium–High	High
Digging new drains or moats around the house or compound	N/A	Medium–High	Medium
Raising the height of the house on wooden stilts or concrete blocks (including livestock coops)	High	High	High
Building flood walls or barriers (e.g. tyres, stones, wooden logs, or coconut husk fences)	Low–Medium	Medium	Medium
Raising the ground level of the compound or low-lying areas of the settlement by adding soil, sand, wood, debris, iron roof sheets, and stones	Low–Medium	High	Medium–High
Relocating the entire house to higher ground	High	High	High
Burying iron roof sheets, tyres, and coconut husks in soil-filled areas to hold soil and prevent erosion	Low–Medium	Medium	Medium–High
Adapting the natural environment pre- and post-flood			
Removal of trees, long grass, and flowers from the drains and waterways to increase the flow of water and prevent rubbish and debris from becoming caught	N/A	Medium	Low-Medium
Planting grass, taro, flowers, and coconut or sandalwood trees around the house and compound to slow the flow of floodwater and prevent soil erosion	N/A	Low	High
Replanting and restoration of coastal mangroves as natural flood barriers	–	High	High

Structural adaptations were usually performed by kinship groups, and houses were raised in sections, enabling residents to continue living there. In the case of new buildings, it was more common for the wider community to assist, with village heads, religious leaders, local builders, and carpenters playing a critical role. Most of the men in the settlements had carpentry skills that had been taught to them by their fathers. Table 2 provides an overview of the inputs and outcomes of the flood protection measures: specifically, the financial costs, implementation hours, and effectiveness and performance of their flood protection, as garnered through resident interviews and photo ordering activities. Cost, labour, and permanence parameters are detailed in Table 3.

**Table 3:** Flood protection key inputs and outcomes key

Categorisation	Low	Medium	High
Cost ($FJD)	Reported as costing between $1 and $150	Reported as costing between $151 and $400	Reported as cost-intensive and costing between $401 and $2,000
Labour (h)	Reported as taking less than a week to implement	Reported as taking between 1 and 2 weeks to implement	More than 2 weeks to implement
Permanence (effectiveness)	Reported as effective for a single flood event	Reported as effective and requiring semi-regular implementation	Reported as very effective and requiring little additional work

## Discussion

### Enhancing community-based flood adaptation: the interplay of resources and institutional support

In line with existing literature, residents' accounts noted that their adaptive capacity was shaped by the availability of assets, access to essential resources, social capital, and institutional support (Chaskin et al. 2001; Gaillard 2010; Goodman et al. 1998; Kretzmann and McKnight 1993; Magis 2010). Despite political, environmental, and socio-economic challenges, residents displayed resourcefulness and dynamism, investing in and self-organising their networks to enact adaptation strategies (Carrasco and Dangol 2019; UNESCAP 2008; Ortiz and Zarate 2004). The widespread adoption of flood protection measures by residents, with a total of 240 initiatives implemented across the 10 settlements, serves as a testament to this. However, societal dynamics played a crucial role in shaping climate responses within communities (Aldrich and Meyer 2015; Cutter et al. 2008). Key factors influencing residents' capacity to implement flood protection measures included the availability of resources and social capital, with the most effective responses often demanding substantial financial investment and coordinated collective action. Permanent measures were generally seen as the most effective strategies for flood adaptation, a finding consistent with research by Mulligan et al. (2017). However, financial constraints limited their implementation. In this instance, community-based programmes can play a pivotal role in enabling community-driven adaptation measures by functioning as critical facilitators that mobilise resources and provide institutional support (Murtinho et al. 2013).

To this effect, programmes can seek to reduce financial burden on residents by funding flood responses or sourcing low-cost solutions through the facilitation of bulk purchases of materials needed for flood protection, making these adaptations more accessible to a broader segment of the community. However, it is crucial to note that not all external funding support is seen to positively influence adaptive capacity (Murtinho et al. 2013). Instances where funding support is unsolicited have been shown to result in a decline in adaptation, underscoring the critical importance that community-driven requests have on adaptation efforts (Murtinho et al. 2013). Additionally, community-based programmes can serve as vital connectors between communities and governmental or non-governmental organisations, advocating for community needs and assisting residents in navigating bureaucratic processes to access government aid and subsidies (Agarwal et al. 2007; Chumo et al. 2023; Satterthwaite 2012; Subramaniam 2018). However, enabling these shifts requires a significant transformation in the funding and approach of adaptation programmes.

Research and funding, as influenced by academia and financial backers, often prioritise certain adaptation strategies, inadvertently sidelining grassroots knowledge and local priorities (Chavez-Rodriguez and Klepp 2018). Predefined outcomes within funding agreements restrict the flexibility essential for genuinely community-driven initiatives, often dictating project trajectories before they even begin (Cook 2012; Ayres and Forsyth 2009; McNamara 2013; McNamara et al. 2020). To authentically empower community-based programmes, a fundamental shift is required in research and funding paradigms. This includes redefining success metrics and engagement terms to accommodate the dynamic nature of community needs and the unpredictable results of grassroots efforts (Cook 2012; El-Askari et al. 1998; Minkler et al. 2003).

Adopting flexible, adaptive funding models that allow for real-time adjustments based on community feedback and changing conditions is crucial (Cook 2012; El-Askari et al. 1998; Minkler et al. 2003). Furthermore, governance structures that recognise and integrate the complexities of social contexts and diverse sources of knowledge are more likely to support transformational adaptation efforts (Few et al. 2007). This approach not only ensures that adaptation strategies are genuinely responsive to local contexts, but also enhances the impact and relevance of projects, fostering a collaborative partnership between funders and communities that is essential for the sustainability and scalability of these initiatives (Chavez-Rodriguez and Klepp 2018; Hay and Mimura 2013; McNamara et al. 2020). Such inclusive and participatory governance models promote community-driven adaptation strategies, empowering communities by ensuring their voices and needs lead the adaptation efforts.

### Redefining effectiveness: embracing diverse community-driven adaptation strategies

In an effort to overcome resource limitations, residents implemented a variety of interconnected measures. The selection and integration of these flood protection measures were guided by several key factors: including local environmental knowledge. This knowledge, based on a nuanced understanding of climatic conditions, weather patterns, and environmental cues, informed the strategic timing of the measures to align with specific stages of flood events, taking into account their speed and magnitude. These findings highlight the interconnected and reciprocal influence of adaptation measures and reinforce the necessity of viewing responses not as isolated interventions, but as integral components of a cohesive whole (Béné et al. 2014; Hertel and Rosch 2010). It further highlights the vital need for community-based programmes to facilitate the development of a variety of context-specific solutions, aligning with narratives that ‘one size doesn’t fit all’ (McNamara 2013, p. 402). Further research is warranted to explore how the collaborative development of more accessible measures can enhance the adaptive capacity. This investigation could provide valuable insights into whether the development of more accessible adaptation measures can progressively strengthen the adaptive capacity as a whole and spill over to the implementation of longer-term adaptation measures.

In line with a common critique of community-based adaptation strategies, residents’ responses predominantly focused on immediate threats. Although these adaptations were incremental and might be perceived by some as mere 'coping strategies' or indicators of 'lack of resilience' (Birkmann et al. 2013, p. 200), residents viewed their flood protection efforts as effective, reporting that their flood protection measures enhanced their families' and community's safety and well-being, by reducing or mitigating exposure to floodwater and subsequent diseases (such as scabies, asthma, and diarrhoea). They also continuously learnt from and adapted to their changing environment, refining their responses accordingly. Due to the highly specific context of adaptation, arriving at a universal definition of effective adaptation presents significant challenges (Morgan et al. 2019; Ngwadla and El-Bakri 2016). In line with the perspective offered by Norris et al. (2008), this study underscores that adaptation should be seen as a process of change, rather than an enhancement of quality, effectiveness, or character beyond current functioning.

Adding to this complexity, there is an increasing demand for a thorough scrutiny of how adaptation is framed and the strategies selected for implementation (Chavez-Rodriguez and Klepp 2018; Wise et al. 2014). The dominant definitions, framing, and measurement of adaptation have largely originated from international bodies in the Global North, which have been criticised for favouring Western and natural sciences over Indigenous and social sciences, reflecting geographical and gender biases among their choice of experts (Beck and Mahony 2018; Gustafsson and Berg 2020; Mach et al. 2014; Mahony 2014; Nyamwanza and Bhatasara 2015; Vardy et al. 2017). This critique emphasises the need to include diverse perspectives, especially from the Global South, to ensure that adaptation and resilience strategies are inclusive, culturally sensitive, and tailored to the specific challenges of various global communities. Western concepts of adaptation, which are far from neutral, often dictate what is considered acceptable and effective (Wise et al. 2014). These concepts typically emphasise technical solutions and legal frameworks, focusing on infrastructure and preventing settlement in high-risk areas (Boyd 2017). Consequently, if community-based programmes adopt Western-centric approaches to adaptation, they may inadvertently dismiss existing community responses as inferior or ineffective (Chavez-Rodriguez and Klepp 2018). Worse still, they could exacerbate social inequalities in informal settlements, as power imbalances and limited access to political support and resources further marginalise these communities (Subramaniam 2018). This risks reinforcing the narrative that marginalised groups in the Global South lack adaptive capacity, thereby impeding residents from enhancing their adaptive capabilities and implementing self-determined flood response strategies. This potential oversight underscores the need for vigilance against replicating social inequities and recognising the inherent value in community-driven initiatives in community-based programmes, even when these may not align with Western expectations of ‘effectiveness’ (Chavez-Rodriguez and Klepp 2018; Subramaniam 2018; Wise et al. 2014). This recognition is crucial, as it aligns with the growing calls for incorporating community perspectives to ensure that adaptation strategies effectively address unique local challenges (Chavez-Rodriguez and Klepp 2018). By valuing the adaptive processes developed within communities, programmes can foster a more inclusive approach that not only acknowledges, but also leverages local insights and knowledge.

### Enhancing adaptation programmes: understanding community dynamics and integrating local and Indigenous knowledge

The importance of understanding and engaging with local and Indigenous knowledge was further exemplified by residents’ strong understanding of the factors that influenced their adaptive capacity, namely resource availability, social networks and dynamics, local and traditional knowledge, shared beliefs, and formal leadership: findings consistent with existing literature (Dangol and Carrasco 2019; Hagedoorn et al. 2019; Islam, et al. 2018; Lebel 2012; McNamara et al. 2020; Ostovar 2019). The intergenerational transmission of knowledge and community collaboration were seen to be pivotal in the selection and implementation of flood responses, with valuable insights into flood responses being disseminated by community elders and leaders of kinship groups. Communal values, traditional social structures, and robust community kinship bonds helped preserve and pass on the cumulative knowledge of flood risks. Moreover, the combination of historical knowledge and residents’ strong affinity with their surroundings was seen to be instrumental in their ability to gauge risk and implement flood protective measures (Lebel 2012). However, these factors primarily influenced the implementation of flood measures within specific segments of the community, such as kinship groups and neighbouring households.

While formal leadership, including village heads and community, and religious and council leaders, was reported to encourage community cooperation and implementation, this predominantly occurred during high-risk events that necessitated rapid community mobilisation for evacuation. However, the implementation of community-wide flood protection measures was impeded by the presence of diverse religious groups, lack of cohesive leaders, and influx of new residents. In this context, the definition of community varies with context, which presents unique challenges for community-based adaptation due to its bottom-up approach (Chung 2023). Effective adaptation is seen as a community-led process that involves co-producing strategies and ensuring diverse stakeholder participation in decision-making (McNamara and Buggy 2017; Singh et al. 2021). However, findings from this study indicate that heterogeneity within communities is not uniform, which introduces additional intra-community challenges.

In this context, if the role of researchers, institutions, and governments are ‘facilitators’, it emphasises the critical importance of conducting governance, epistemology, and social network analysis at the inception of community-based adaptation programmes (McNaught 2024; Ostovar 2019). This perspective acknowledges that adaptation is not apolitical; rather a process that requires power structures to be contested, negotiated or reinforced (Brink et al. 2023; Chu 2018; Eriksen et al. 2015; IPCC 2022; Manuel-Navarrete et al. 2011; Woroniecki et al. 2020). Integrating this analysis can deepen our understanding of community structures, power dynamics, and the inclusion of diverse perspectives, ensuring that external actors are deeply aware of the structural dynamics at play and can serve as a vehicle for social development rather than reinforcing social inequalities (Ensor et al. 2018; Subramaniam 2018). This approach can facilitate the early identification of potential barriers and enablers of community adoption, providing greater insights into how external actors can support community-wide adaptation efforts. This study highlights the critical role of focusing facilitation efforts on local social structures and belief systems. However, further research is recommended to avoid paternalistic approaches and maintain sensitivity to the community's cultural dynamics, customs, values, and norms (Ayers and Forsyth 2009; McNamara et al. 2020; Westoby et al. 2019).

## Conclusion: strengthening community-based adaptation through inclusive strategies

This study examining how residents of urban informal settlements in Fiji adapt to flooding represents one component of a much larger puzzle. Although communities showed strong adaptive capacity with the implementation of over 240 flood protection measures, the determinants by which residents adapt were complex and often determined by broader societal dimensions—the most influential being resource availability and social capital. Findings indicate the importance of viewing adaptation as a process of change (Norris et al. 2008) and underscore the crucial role that community-based programmes can play in enhancing existing community measures and collaborating with communities to develop a range of solutions tailored to their specific needs. The study outlines the importance of valuing local insights and initiatives and mitigating Western-centric approaches to adaptation that may undervalue local responses and reinforce stereotypes of lack of adaptive capacity, which often impede self-determined strategies (Chavez-Rodriguez and Klepp 2018; Cook 2012; Ayres and Dodman 2010; McNamara 2013; McNamara et al. 2020). To achieve this, in-depth engagement with communities is necessary to understand their existing practices and the factors influencing adaptation. This in-depth engagement requires a fundamental shift in research and funding to truly empower community-driven adaptation programmes, ensuring they are flexible, responsive to local needs, and capable of integrating diverse knowledge sources (Few et al. 2007; Chavez-Rodriguez and Klepp 2018; Hay and Mimura 2013; McNamara et al. 2020).

In the realms of community-based adaptation, this study suggests a number of avenues for greater facilitation of community-driven adaptation. For financial support, community programmes can play a critical role in working with communities to identify the areas of funding support and seeking to lessen the financial burden on residents by funding community-driven flood responses or facilitating bulk purchases of materials, making adaptation more widely accessible (Chavez-Rodriguez and Klepp 2018). For social capital, this study highlights the significance of social networks and traditional knowledge in shaping community responses to flooding. It also identifies that the heterogeneity within communities varies, presenting unique intra-community challenges (Chung 2023). The findings advocate for a community-led adaptation process, emphasising that effective strategies should be co-produced with diverse stakeholder participation (McNamara and Buggy 2017; Singh et al. 2021). Moreover, there is a crucial need for community-based programmes to thoroughly understand community dynamics and power structures to facilitate effective adaptation efforts and prevent paternalistic interventions (Ensor et al. 2018; McNaught 2024; Ostovar 2019).

Future research is needed to better understand the effectiveness of the adaptation strategies that communities choose to implement (Murtinho and Hayes 2012). This study focuses on current adaptation measures, not the outcomes. While assessing the process of adaptation can reveal the factors influencing these initiatives, further research is required to evaluate the effectiveness of these measures and the role that external institutions can play in helping residents enhance these strategies. It is equally important that further research be conducted to develop effective strategies for preventing paternalistic approaches and maintaining sensitivity to the community's cultural dynamics, customs, and values and norms (Ayers and Forsyth 2009; McNamara et al. 2020; Westoby et al. 2019).
